# Defining valid breeding goals for animal breeds

**DOI:** 10.1186/s12711-023-00855-6

**Published:** 2023-11-21

**Authors:** Robin Wellmann, Nicolas Gengler, Jörn Bennewitz, Jens Tetens

**Affiliations:** 1 Department of Animal Genetics and Breeding, University of Hohenheim, 70599 Stuttgart, Germany; 2 TERRA Teaching and Research Center, University of Liège, Gembloux Agro-Bio Tech, 5030 Gembloux, Belgium; 3 Department of Animal Sciences, Georg-August-University Göttingen, 37077 Göttingen, Germany

## Abstract

**Background:**

The objective of any valid breeding program is to increase the suitability of a breed for its future purposes. The approach most often followed in animal breeding for optimizing breeding goals assumes that the sole desire of the owners is profit maximization. As this assumption is often violated, a generalized approach is needed that does not rely on this assumption.

**Results:**

The generalized approach is based on the niche concept. The niche of a breed is a set of environments in which a small population of the breed would have a positive population growth rate. Its growth rate depends on demand from prospective consumers and supply from producers. The approach involves defining the niche that is envisaged for the breed and identifying the trait optima that maximize the breed’s adaptation to its envisaged niche within the set of permissible breeding goals. The set of permissible breeding goals is the set of all potential breeding goals that are compatible with animal welfare and could be reached within the planning horizon of the breeding program. In general, the breed’s adaptation depends on the satisfaction of the producers with the animals and on the satisfaction of the consumers with the products produced by the animals. When consumers buy live animals, then the breed needs to adapt to both the environments provided by the producers, and the environments provided by the consumers. The profit function is replaced by a more general adaptedness function that measures the breed’s adaptation to its envisaged niche.

**Conclusions:**

The proposed approach coincides with the traditional approach if the producers have the sole desire to maximize their income, and if consumer preferences are well reflected by the product prices. If these assumptions are not met, then the traditional approach to breeding goal optimization is unlikely to result in a valid breeding goal. Using the example of companion breeds, this paper shows that the proposed approach has the potential to fill the gap.

## Background

The desired performance, anatomy, behaviour, and environmental impact of a breed can be called its breeding goal. Although domestic animals are selected to match specific breeding goals, the validity of their breeding goals is often unclear, especially for breeds that are not kept for profit.

In general, a breeding goal can be said to be valid, if selection of the breed towards the breeding goal increases the general suitability of the breed for its anticipated future purposes, and if the number of animals used for these purposes remains sufficiently large for the long-term survival of the breed. The number of animals required depends on the average number of offspring per breeding animal. The population size should be large enough so that a breeding program can be implemented that maintains an effective population size of at least 100 and allows genetic progress to be made [1].

Improving the suitability of a breed for its future purposes increases its competitiveness against other breeds, which results in an increase or maintenance of the population size. Consequently, a breeding goal is valid if a set of environments exists in which a population of sufficient size that matches the breeding goal would have a reproduction rate greater than or equal to its death rate. This set of environments can be called the breed’s future niche. The validity requirement can, therefore, be reformulated by saying that a breeding goal is valid if a sufficiently large niche exists for a breed with the envisaged phenotype, and if this niche continues to exist in the future.

The concept of the ecological niche was already introduced in 1917 by Grinnell [2], but it took four decades until Hutchinson provided a concise definition [3]. According to this definition, a niche is an $n$-dimensional hypervolume, where the dimensions are environmental conditions that define the requirements for a population to persist. Thus, the niche of a species is a set of environments in which the birth rate of a sub-population that is placed into the environment is equal to or greater than its death rate. The niche concept extends to domestic animal breeds. The niche of a species depends on its performance, anatomy and behaviour. While a recurring theme in ecology is the characterization of the niche of a species with a given performance, anatomy and behaviour, the priority set by animal breeders is reversed. Here, the challenge is to find the optimum performance, anatomy and behaviour that guarantee that a given set of environments is part of a breed’s niche. Animal breeders would then set up a breeding program that causes the breed’s anatomy and behaviour to converge towards the optimum.

A main difference between wild species and domestic breeds is that domestic animals have owners while wild species have not. The owners provide the environments of the breeds, i.e., they provide specific conditions to which breeds may adapt and are simultaneously the resources for which the breeds compete. For domestic animals, the space $\mathcal{E}$ of environments characterizes the desires that the owners might associate with owning an animal from the species and the values of influencing variables such as market prices of required resources. Hence, a vector $\mathbf{e}\in \mathcal{E}$ provides values for all factors that might influence whether an owner who provides a specific environment prefers a given animal over others. The niche ${\mathcal{E}}_{b}\subset \mathcal{E}$ of breed $b$ characterizes all potential owners that would prefer the breed over other breeds.

Let ${{\varvec{\upmu}}}_{b}$ denote the parameter of the phenotypic distribution of breed $b$. The adaptedness $a\left({{\varvec{\upmu}}}_{b},\mathbf{e}\right)$ of breed $b$ to environment $\mathbf{e}$ can be defined as a function of the breed’s population growth rate. The reason for a positive growth rate is usually that the owners like the breed or its performance. Therefore, $a\left({{\varvec{\upmu}}}_{1},\mathbf{e}\right)>a\left({{\varvec{\upmu}}}_{2},\mathbf{e}\right)$ means that a breed with phenotypic distribution ${{\varvec{\upmu}}}_{1}$ would be preferred over a breed with phenotypic distribution ${{\varvec{\upmu}}}_{2}$ by owners who provide environment $\mathbf{e}$. Accordingly, $a\left({{\varvec{\upmu}}}_{b},{\mathbf{e}}_{1}\right)>a\left({{\varvec{\upmu}}}_{b},{\mathbf{e}}_{2}\right)$ means that people providing environment ${\mathbf{e}}_{1}$ like the breed more than people providing environment ${\mathbf{e}}_{2}$. The term $a\left({{\varvec{\upmu}}}_{b},\mathbf{e}\right)$ could alternatively be called the attractiveness of the breed for owners who provide environment $\mathbf{e}$, or the satisfaction of the owners with the breed. While the current niche of breed $b$ is the set of all environments $\mathbf{e}\in \mathcal{E}$ for which $a\left({{\varvec{\upmu}}}_{b},\mathbf{e}\right)\ge a\left({{\varvec{\upmu}}}_{b{\prime}},\mathbf{e}\right)$ for all breeds $b{\prime}$, the term ‘envisaged niche’ refers to the niche the breed should occupy in the future.

Averaging the breed’s adaptedness to the environments that are included in the breed’s envisaged niche ${\mathcal{E}}_{b}$ provides the adaptedness ${a}_{t}\left({{\varvec{\upmu}}}_{b},{\mathcal{E}}_{b}\right)$ of the breed to that niche. That is:

1 $${a}_{t}\left({{\varvec{\upmu}}}_{b},{\mathcal{E}}_{b}\right)={\mathbb{E}}\left(a\left({{\varvec{\upmu}}}_{b},{\mathbf{e}}_{t}\right)|{\mathbf{e}}_{t}\in {\mathcal{E}}_{b}\right),$$

where the random vector ${\mathbf{e}}_{t}\in \mathcal{E}$ is the environment of an animal from the species that is randomly chosen at time $t$. Because vector ${\mathbf{e}}_{t}$ describes the preferences of a randomly chosen owner, and because people change their preferences over time, the probability distribution of vector ${\mathbf{e}}_{t}$ also changes with time. We call the term ${a}_{t}\left({{\varvec{\upmu}}}_{b},{\mathcal{E}}_{b}\right)$, when considered as a function of the first argument ${{\varvec{\upmu}}}_{b}$, the adaptedness function of the breed.

Optimizing the breeding goal of a breed means defining an envisaged niche ${\mathcal{E}}_{b}$ for the breed and finding the optimum intermediate breeding goal ${\dot{{\varvec{\upmu}}}}_{b}^{i}$ that maximizes the adaptedness function ${a}_{t}\left({{\varvec{\upmu}}}_{b},{\mathcal{E}}_{b}\right)$ on a set ${\mathcal{U}}_{b}$ of permissible breeding goals. In particular, a breeding goal would be inadmissible, if a change in genetic variances and covariances reduces the genetic variance in the selection index to zero before the breeding goal could be achieved. The term ‘breeding goal’ is often used in the literature to denote the so-called ‘aggregate genotype’ [4], while this paper distinguishes both terms: an aggregate genotype scores animals from the current generation, while a breeding goal defines a desired intermediary state of a breeding program.

The traditional approach to breeding goal optimization is based on profit calculations [5]. In this case, the phenotypic covariance matrix is treated as a constant, and ${{\varvec{\upmu}}}_{b}$ is the vector of trait means. The profit function $\phi \left({{\varvec{\upmu}}}_{b}\right)$ computes the expected monetary profit of a breed with trait means ${{\varvec{\upmu}}}_{b}$ for a certain production system. For a breeding program with a planning horizon that lasts until time ${t}_{1}$, the calculation of the optimum selection index requires knowledge of the local maximum ${\dot{{\varvec{\upmu}}}}_{b}^{i}$ of the profit function in the response area ${\mathcal{R}}_{b}$ [6, 7]. The response area is the set of all putative breeding goals that can be reached until time ${t}_{1}$. The selection index can then be calculated with the desired gain approach [8]. The vector with desired gains equals $\Delta {\varvec{\upmu}}={\dot{{\varvec{\upmu}}}}_{b}^{i}-{{\varvec{\upmu}}}_{b}^{c}$, where ${{\varvec{\upmu}}}_{b}^{c}$ is the vector of current trait means. The approach could be further improved by discounting future genetic gains [9], or by relaxing simplistic assumptions about the trait architecture [10], which is, however, rarely done in practice.

The approach proposed in this paper adheres to the traditional approach described above, but replaces the profit function $\phi \left({{\varvec{\upmu}}}_{b}\right)$ with the adaptedness function ${a}_{t}\left({{\varvec{\upmu}}}_{b} ,{\mathcal{E}}_{b}\right)$, and the response area ${\mathcal{R}}_{b}$ with a set ${\mathcal{U}}_{b}$ of permissible breeding goals. Although generalizing the traditional approach to breeding goal optimization is straightforward, the application of niche theory to animal breeding faces several challenges. First, it is unclear what the relevant dimensions of a breed’s niche are, second, it is unclear how the optimum phenotype of a breed depends on the niche, and third, it is unclear how to perform the optimization in practice. The aim of this study was to resolve these issues. Frequently used symbols are summarized in Table 1.

**Table 1:** List of frequently used symbols

Symbol	Explanation
General	
$\mathcal{Y}$	Space of phenotypes
Set of environments	
$\mathcal{E}$	Space of environments
${\mathcal{E}}_{b}^{c}\subset \mathcal{E}$	Current niche of breed $b$
${\mathcal{E}}_{b}\subset \mathcal{E}$	Potential niche of breed $b$
$\mathcal{D}$	Space of owner desires. Each component of $\mathcal{D}$ assesses the extent to which the animal should be suitable for satisfying a certain desire
$\mathcal{V}$	Space of influencing variables
Potential breeding goals	
$\mathcal{P}$	Set of potential breeding goals
${\mathcal{S}}_{b}\subset \mathcal{P}$	Search area in which the optimum breeding goal of breed $b$ is searched
${\mathcal{U}}_{b}\subset {\mathcal{S}}_{b}$	Set of permissible breeding goals for breed $b$
${{\varvec{\upmu}}}_{tb}\in \mathcal{P}$	State of the breeding program of breed $b$ at time $t$
${{\varvec{\upmu}}}_{b}^{c}\in \mathcal{P}$	Current state of the breeding program
${{\varvec{\upmu}}}_{b}\in \mathcal{P}$	Putative breeding goal of breed $b$
${\dot{{\varvec{\upmu}}}}_{b}^{i}\in {\mathcal{U}}_{b}$	Optimum intermediate breeding goal of breed $b$
${\dot{{\varvec{\upmu}}}}_{b}^{s}\in {\mathcal{S}}_{b}$	Optimum breeding goal of breed $b$ in the search area
Key parameters	
${\mathfrak{m}}_{t}\left({\mathcal{E}}_{b}\right)$	Expected size of the breed’s envisaged niche ${\mathcal{E}}_{b}$ at time $t$
$a\left({{\varvec{\upmu}}}_{b},\mathbf{e}\right)$	Adaptedness of breed $b$ to environment $\mathbf{e}\in \mathcal{E}$
${a}_{t}\left({{\varvec{\upmu}}}_{b},{\mathcal{E}}_{b}\right)$	Adaptedness of breed $b$ to its envisaged niche ${\mathcal{E}}_{b}\subset \mathcal{E}$ at time $t$
${\mathrm{TM}}_{\mathbf{e}}\left(\mathbf{y}\right)$	Total merit of an animal with phenotype vector $\mathbf{y}$ in environment $\mathbf{e}$
${\phi }_{\mathbf{v}}\left(\mathbf{y}\right)$	Monetary profit the owner has from keeping an animal with phenotype $\mathbf{y}$
${\mathrm{R}}_{\mathbf{e}}\left(\mathbf{y}\right)$	Non-monetary reward the owner perceives he has
${\mathrm{C}}_{\mathbf{e}}\left(\mathbf{y}\right)$	Non-monetary costs the owner perceives he has
${\alpha }_{p}$, ${\alpha }_{n}$,${\alpha }_{c}$	Weights given to the total merits of an animal and its products for the producers, owners, and consumers, respectively
${\lambda }_{\mathbf{e}}$	Weight given to non-monetary rewards and costs

## Theory

This section proposes a general framework for the optimization of breeding goals of animal breeds. The space $\mathcal{E}$ of environments is defined and its dimensions are examined. The space $\mathcal{P}$ of potential breeding goals and the adaptedness of a breed with state ${{\varvec{\upmu}}}_{b}\in \mathcal{P}$ to an environment $\mathbf{e}\in \mathcal{E}$ are defined. The problem of optimizing breeding goals is identified as the problem of maximizing the adaptedness of a breed to its envisaged niche, and a general method for breeding goal optimization is proposed.

### The space $\mathcal{E}$ of environments

The set $\mathcal{E}$ of environments is a high-dimensional vector space. The dimensions of this vector space are the factors that can influence the competitiveness of a breed in a given environment. All factors are included that affect whether a subpopulation of the breed that is placed into a particular environment will survive, or decrease in size and eventually become extinct. Thus, the values these factors take in a vector $\mathbf{e}\in \mathcal{E}$ define conditions to which a breed could adapt [11].

The space of environments consists of different subspaces that can be combined by the Cartesian product “$\times$” to create the whole space. These subspaces are different for wild animal species and domestic breeds. This is because the population size of a wild animal species is determined by the values taken by the relevant biotic and abiotic factors, while the population size of a domestic animal breed depends also on owner-related factors. For domestic animal breeds, the space of environments is written as $\mathcal{E}=\mathcal{D}\times \mathcal{V}$, where subspace $\mathcal{D}$ assesses all desires, an owner might associate with owning an animal, and subspace $\mathcal{V}$ includes all influencing variables that affect how an owner evaluates the suitability of an animal to satisfy a certain desire. The relevant biotic and abiotic factors are included in subspace $\mathcal{V}$. Abiotic factors include temperature, climate, and soil type, while biotic factors include feed quality and the presence of pathogens.

For example, for a commercial dairy cattle breed that is mainly kept for profit, subspace $\mathcal{D}$ could assess the extent to which the owner wants to make profit and the extent to which he wants his breed to be easy-going, while subspace $\mathcal{V}$ includes, among other parameters, the milk price, and the feed quality. Feed quality would be included because a high-yielding breed would not be considered easy-going on a farm with low-quality feed and it also provides a lower profit on that farm.

We call the dimensions of $\mathcal{E}$ the dimensions of ownership. The dimensions of ownership characterize people who own an animal from the species as well as people who do not own an animal, but might decide to do so. Different owners score differently in the different dimensions of ownership. Therefore, they could provide different niches for their animals. A diversification of breeds arises when different breeds specialize to fit the needs of different owners.

**Fig. 1:**
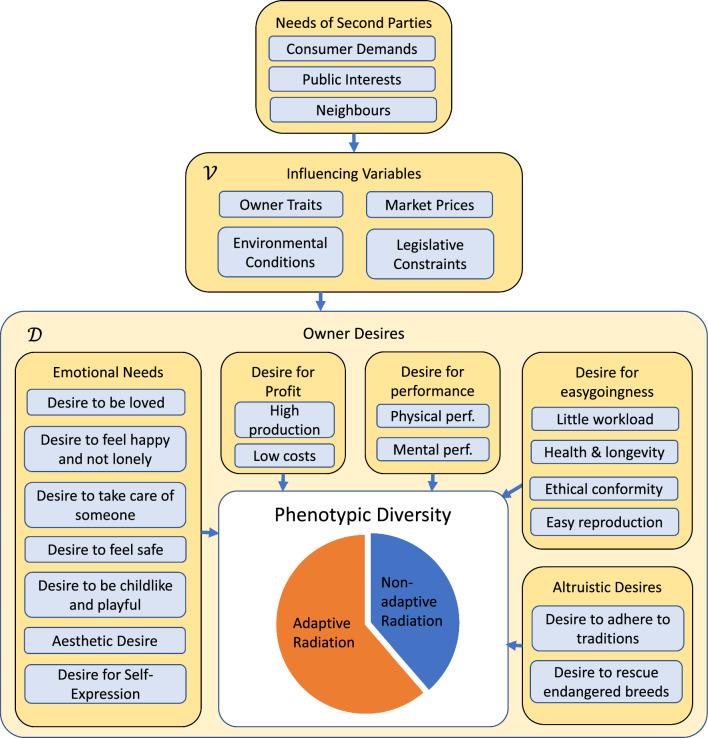
Illustration of the space $\mathcal{E}$ of environments. Each environment $\varvec{\mathrm{e}}=\left(\varvec{\mathrm{d}},\varvec{\mathrm{v}}\right)$ contains a subvector $\varvec{\mathrm{d}}\in \mathcal{D}$ that quantifies the extents to which the owner wants his animal to satisfy certain potential desires. The suitability of a breed to satisfy a certain desire depends on the subvector $\varvec{\mathrm{v}}\in \mathcal{V}$ of influencing variables. Each desire that an owner could associate with owning an animal can have implications for the optimum phenotype and the phenotypic diversity of the species. Thus, it can lead to adaptive or non-adaptive radiation. The figure illustrates what the influencing factors are

The space $\mathcal{D}$ of owner desires and the space $\mathcal{V}$ of influencing variables, which are illustrated in Fig. 1, are presented in detail below. We formalize the definition of the space $\mathcal{D}$ of owner desires and the space $\mathcal{V}$ of influencing variables, generalizing non-formal attempts already found in the literature [12–15].

#### The space $\mathcal{D}$ of owner desires

The space $\mathcal{D}$ of owner desires can be decomposed as:

2 $$\mathcal{D}={\mathcal{D}}_{\mathrm{profit}}\times {\mathcal{D}}_{\mathrm{performance}}\times {\mathcal{D}}_{\mathrm{emotional }}\times {\mathcal{D}}_{\mathrm{easygoing}}\times {\mathcal{D}}_{\mathrm{altruistic}},$$

where each subspace corresponds to a certain category of desires. The subspace assesses the extents to which the owner would like the animal to be able to satisfy desires from that category. The categories are described below.

*Desire to make profit* The one-dimensional subspace ${\mathcal{D}}_{\mathrm{profit}}$ quantifies the importance an owner places on having high profit. The desire for profit can be satisfied by productive animals and by animals that cause little monetary costs. Owners of livestock breeds are likely to score high in this subspace. The traditional approach to breeding goal optimization only takes profit-related desires into account, in which case $\mathcal{D}={\mathcal{D}}_{\mathrm{profit}}$. In practice, however, other desires might be of similar importance for ownership decisions. For example, a good working atmosphere might cause people to be satisfied with less income. Being surrounded by good-looking animals can contribute to a good working atmosphere. One dimension of ownership should, therefore, access the owner’s desire to be surrounded by good looking animals. Consequently, the traditional approach is unsatisfactory even for livestock breeds.

*Performance-related desires* Subspace ${\mathcal{D}}_{\mathrm{performance}}$ measures the extent to which the owner wants his animals to have a good physical and mental performance in everyday life and when they are used for specific activities. For horses, possible activities include horse racing, show-jumping, eventing, dressage, and performing certain gaits. For dogs, possible activities include taking part in agility competitions, being a guide for the blind, a specialized hunting dog, a rescue dog, a sledding dog, or a herding dog. Owners of working dogs and horses are likely to score high in this subspace.

*Desire to get emotional support* Subspace ${\mathcal{D}}_{\mathrm{emotional}}$ assesses the extent to which the animal should satisfy certain human emotional needs and desires. A human need arises when a person is in a state of deprivation and reflects the desire of the person to get into the associated positive emotional state. Many human needs can be satisfied by owning animals, which includes the need to be loved, the need to feel happy and not lonely, the need to take care of someone, the need to feel safe, and the need to be childlike and playful [16]. Further emotional needs are to have an animal of high aesthetic value, and the desire to express one's own personality and lifestyle through owning certain animals. Each dimension of this subspace assesses the importance that an owner attaches to the animal's ability to satisfy a particular need or desire. As detailed below, emotional needs can be satisfied by animals whose conformation and behaviour activates certain pattern recognition schemes in the human brain. Owners of social support animals are likely to score high in these dimensions. Although companion animals are especially suitable for satisfying human emotional needs, livestock breeds also offer opportunities. For example, hobby farmers might prefer good-looking breeds with little or no escape distance, or they might prefer breeds with a certain look, such as Highland cattle, to emphasize their country-style way of life.

*Desire for easygoingnes.* Subspace ${\mathcal{D}}_{\mathrm{easygoing}}$ assesses the desire of avoiding complications that could arise from owning an animal. This general desire can be split into several specific desires, such as the desire to have little monetary costs and workload, the desire that the measures that are to be taken do not exceed the owner’s qualification, the desire for ethical conformity, the desire to own healthy and long-living animals, and the desire that the breed can easily reproduce. The desires assessed in this subspace are secondary desires in the sense that they arise only after the decision to own an animal has been made. They are secondary desires because it would be most easy-going for most people to own no animal at all. The desire for easygoingness can, therefore, not be the sole reason for owning an animal.

*Altruistic desires* Subspace ${\mathcal{D}}_{\mathrm{altruistic}}$ assesses the desires that people may have even though they have no personal benefit from satisfying them. We call these desires ‘altruistic’. For example, people might feel the need to adhere to traditions, in which case they could continue to keep the same breed as their parents and prefer the traditional breed type. In addition, people might feel the need to rescue endangered breeds. The desire of rescuing endangered breeds causes the phenomenon that the rareness of a breed can increase the demand for that breed, which implies that the reproduction rate of an endangered breed could stabilize at a low level. The existence of these desires implies that the exclusion principle in ecology does not apply to domestic breeds. That is, different domestic breeds can coexist in the same niche. This group of desires contributes, therefore, to the genetic variation of a species.

#### The space $\mathcal{V}$ of influencing variables

The space $\mathcal{V}$ of influencing variables includes factors that affect how a person evaluates the ability of an animal to satisfy a certain desire. The space can be decomposed as:

$$\mathcal{V}={\mathcal{V}}_{\mathrm{Owner}}\times {\mathcal{V}}_{\mathrm{Physical\> Env}.}\times {\mathcal{V}}_{\mathrm{Market}}\times {\mathcal{V}}_{\mathrm{Legislative}},$$

where each subspace corresponds to a certain category of influencing variables. The categories are described below.

*Owner traits* Subspace ${\mathcal{V}}_{\mathrm{Owner}}$ of owner traits assesses personality traits and other characteristics of the owner, such as the owner’s personality, fitness, allergies, intelligence, income, and habitual behaviour, but also whether the person is a breeder. Owner traits can affect the breed choice. In particular, owner traits affect the extent to which complications can be handled or tolerated by the owner.

*Physical environment* Subspace ${\mathcal{V}}_{\mathrm{Physical\> Env.}}$ characterizes the physical environment that the owner provides for his animals. The physical environment of a livestock breed is the farm, while the physical environment of companion animals is the home and garden of the owner. Each dimension of this subspace quantifies an aspect of the physical environment that can affect the favoured phenotype. Examples for dairy cattle are the temperature, climate, soil quality, feed quality, the lengths of the stalls, and the presence of a milking robot. Examples for dogs are the presence of noise-sensitive neighbours and the presence of a fenced garden.

*Market prices* Subspace ${\mathcal{V}}_{\mathrm{Market}}$ provides market prices for products and resources. The costs of the required resources, and the market prices of the products produced by the animal affect the profit of a breed. Examples are the feed costs, and the milk price of dairy cattle.

*Legislative framework conditions* Subspace ${\mathcal{V}}_{\mathrm{Legislative}}$ characterizes legislative framework conditions. Legislative framework conditions assess the existence of a certain law, the expected time until commencement of an anticipated law that can restrict ownership decisions, and the monetary values of incentives and deterrents that are foreseen by law for influencing an owner’s behaviour. Well-designed laws enable the legislation to canalize the interests of the owners to the benefit of the public. Examples for legislative framework conditions with implications on breeding goal optimization are dangerous dog acts, animal welfare regulations, taxes on emissions, emissions certificates, and subsidies for keeping local breeds.

### The space $\mathcal{Y}$ of phenotypes

The phenotype, also phenome, of an animal can be described by a vector with trait values, so the space of phenotypes is a set $\mathcal{Y}\subset {\mathbb{R}}^{K}$ with $K$ being the number of traits. The set of traits used for defining a phenotype should include all traits that can affect ownership decisions. Since it is often not clear a priori what these traits are, the space of phenotypes could be defined to include more than the required traits. The space can be decomposed as:

$$\mathcal{Y}={\mathcal{Y}}_{\mathrm{Prod}.}\times {\mathcal{Y}}_{\mathrm{Perf}.}\times {\mathcal{Y}}_{\mathrm{Conf}. \&\mathrm{ Mov}.}\times {\mathcal{Y}}_{\mathrm{Behaviour}}\times {\mathcal{Y}}_{\mathrm{Functional}}\times {\mathcal{Y}}_{\mathrm{Suppl}.},$$

where each subspace corresponds to a certain category of traits. The categories are detailed below. A definition of each trait category is given, and examples are provided.

*Production traits* Subspace ${\mathcal{Y}}_{\mathrm{Prod.}}$ includes the production traits, which measure the quantity or quality of the products that are produced by the animal. Some products are valued by the consumers while others are penalized by the legislation. Examples for the former are the milk-yield of dairy cattle, the egg-count of layer chickens, and the meat quality of pigs. Production traits that might be penalized by the legislation are environmental impact traits such as the methane production of ruminants. The litter size could be considered a production trait if the consumers buy live animals.

*Performance traits* Subspace ${\mathcal{Y}}_{\mathrm{Perf.}}$ includes performance traits, which measure the physical and mental performance of the animals when they are used for specific activities, whereby production traits are excluded. Examples for performance traits include the performance of a horse in horse jumping competitions, or the performance of a dog who is used as a guide for the blind.

*Conformation and movement traits* Subspace ${\mathcal{Y}}_{\mathrm{Conf. \& Mov.}}$ includes conformation and movement traits. Conformation traits, which are also called morphological traits, measure aspects of the form and structure of an animal. This includes aspects of the outward appearance (body measurements, body form, colour), as well as the form and structure of the internal parts, such as bones. Examples for conformation traits are the udder score and the feet-and-leg score of dairy cattle, and the breast muscle development of broiler chicken. A movement trait could measure how well an animal performs a certain gait. In many circumstances, especially for companion animals, it is not possible to know the relevant conformation traits a priori. In these cases, it is appropriate to define the optimum conformation by a physical reference model (i.e., sculpture) that defines the long-term breeding goal for conformation traits. The intermediate breeding goal and the weights of the traits in the selection index can then be identified in a second step.

*Behaviour traits* Subspace ${\mathcal{Y}}_{\mathrm{Behaviour}}$ describes the instinctive behaviour of an animal. A description of a desired instinctive behaviour should reveal how the animal is expected to react to a certain stimulus in a given situation, and how intense the reaction should be. The desired instinctive behaviour could, therefore, best be defined by an ethogram-like document.

*Functional traits* Subspace ${\mathcal{Y}}_{\mathrm{Functional}}$ includes functional traits. Functional traits are those that affect the monetary costs that are associated with keeping the animal. They include longevity, fertility, ease of birth, feed efficiency, and the health status. Functional traits can affect the owner’s satisfaction by satisfying the owner’s monetary desires and by satisfying secondary desires such as the perceived easygoingness of the breed. The relevance of a functional trait for owners can depend on themselves. For example, the relevance for easy births depends on whether the owner intends to breed, and on whether he has experience with difficult births.

*Supplemental traits* Subspace ${\mathcal{Y}}_{\mathrm{Suppl.}}$ includes traits that provide supplemental information such as the animal’s sex, the genotype of the animal at known quantitative trait loci (QTL), and the genotypes at loci carrying disease alleles.

### The total merit of an animal

We consider a breeding scheme where at most two relevant transactions can take place: the owners who provide the envisaged niche for the breed may buy their animals from producers (also called breeders), and they may sell products produced by their animals. The merit of an animal can thus be measured by the satisfaction of the breeder with his animals, by the satisfaction of the owner who bought his animal from a breeder, and by the satisfaction of the consumers who bought the products produced by the animal.

We call the satisfaction of an owner with animal $i$ the total merit ${\mathrm{TM}}_{\mathbf{e}}\left({{\mathbf{y}}}_{i}\right)$ of the animal for the owner. The animal’s total merit depends on the environment $\mathbf{e}$ that the owner offers his animals, and on the phenotype vector ${{\mathbf{y}}}_{i}$ of the animal. Accordingly, the total merit of the animal for the producer is denoted as ${\mathrm{TM}}_{{\mathbf{e}}_{{{p}}}}\left({{\mathbf{y}}}_{i}\right)$, where ${\mathbf{e}}_{{{p}}}$ is the environment, the producer offers his animals. In addition, we denote with $\mathrm{PQ}\left({{\mathbf{y}}}_{i}\right)$ the quality of the final product as rated by the consumers.

Recall that a breeding program should increase or stabilize the population size. The adaptedness $a\left({{\varvec{\upmu}}}_{b},\mathbf{e}\right)$ of breed $b$ to environment $\mathbf{e}$ was, therefore, defined as a function of the breed’s population growth rate. The breed’s population growth rate depends on the satisfactions of the owners and producers with their animals, and on the satisfaction of the consumers with the final product, so it can be defined as:

3 $$a\left({{\varvec{\upmu}}}_{b},\mathbf{e}\right)={\mathbb{E}}\left({\alpha }_{p}{\mathrm{TM}}_{{\mathbf{e}}_{p}}\left(\mathbf{y}\mathbf{^{\prime}}\right)+{\alpha }_{n}{\mathrm{TM}}_{\mathbf{e}}\left({\mathbf{y}}\right)+{\alpha }_{c}\mathrm{PQ}\left(\mathbf{y}\right)\right),$$

where $\mathbf{y}$ is the vector of the phenotype of a randomly chosen animal from the breed that lives in the fixed environment $\mathbf{e}$, and $\mathbf{y}\mathbf{^{\prime}}$ is the vector of phenotype of a randomly chosen animal from the breed that lives in the environment ${\mathbf{e}}_{p}$ provided by the producer. The weights ${\alpha }_{p},{\alpha }_{n},{\alpha }_{c}\ge 0$ with ${\alpha }_{p}+{\alpha }_{n}+{\alpha }_{c}=1$ are chosen depending on what the limiting factor for the population size is.

When breeders do not solely breed for profit, or if the quality of a product is not well reflected by the market prices, then an imbalance between demand and supply can emerge that limits the breed’s population size. The weight ${\alpha }_{c}$ given to the consumer is chosen such that the balance between demand and supply improves over time. It is common in livestock breeding to assume ${\alpha }_{c}=0$ because product quality is assumed to be well reflected by the product prices. The optimum weight ${\alpha }_{c}$ could differ from zero if the consumer preferences are not well reflected by the product prices, or if the breeders do not keep their animals for profit.

**Fig. 2:**
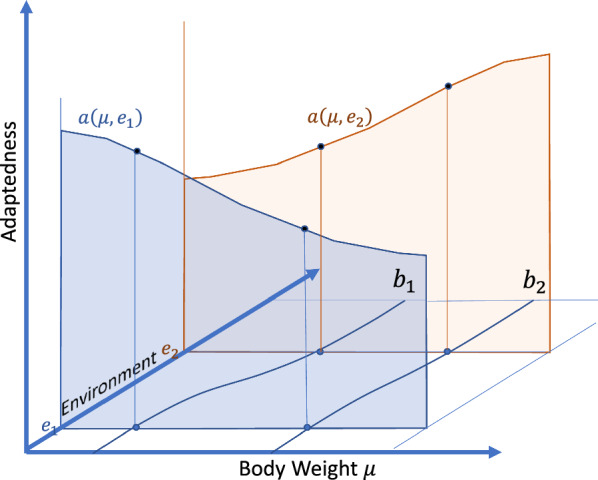
Illustration of the adaptation of a breed to different environments. Illustrative example with a one-dimensional space $\mathcal{E}$ of environments, and a one-dimensional space $\mathcal{Y}$ of phenotypes. The trait of interest is body weight. A small body weight is preferred in environment ${e}_{1}$, while a large body weight is preferred in environment ${e}_{2}$, so breed ${b}_{1}$ is adapted to environment ${e}_{1}$, while breed ${b}_{2}$ is adapted to environment ${e}_{2}$. Note that not only the preferred body weight changes depending on the environment, but also the realized body weight. The animals get heavier in environment ${e}_{2}$, e.g., due to improved feed quality

Random vector $\mathbf{y}$ in Eq. (3) has a probability distribution with parameter ${{\varvec{\upmu}}}_{b}\left(\mathbf{e}\right)$, while random vector $\mathbf{y}\mathbf{^{\prime}}$ has conditionally on ${\mathbf{e}}_{p}$ a probability distribution with parameter ${{\varvec{\upmu}}}_{b}\left({\mathbf{e}}_{p}\right)$. That is, the phenotypic distribution of the animals is allowed to depend on the environment, which broadens the definition of ${{\varvec{\upmu}}}_{b}$ given in the Background section. The dependency of the total merit on both, the environment, and the trait mean is illustrated in Fig. 2.

The owners that offer the niche are often identical with the breeders, which is usually the case in cattle breeding. In this case, ${\mathrm{TM}}_{{\mathbf{e}}_{p}}\left(\mathbf{y}\mathbf{^{\prime}}\right)={\mathrm{TM}}_{\mathbf{e}}\left({\mathbf{y}}\right)$ can be assumed, so the adaptedness function simplifies to:

$$a\left({{\varvec{\upmu}}}_{b},\mathbf{e}\right)={\mathbb{E}}\left({\alpha }_{n}{\mathrm{TM}}_{\mathbf{e}}\left({\mathbf{y}}\right)+{\alpha }_{c}\mathrm{PQ}\left(\mathbf{y}\right)\right).$$

Sometimes, the owners that offer the niche are identical with the consumers, which is usually the case in companion dog breeding. In this case, there is no final product other than the animal itself, so the adaptedness function simplifies to:

$$a\left({{\varvec{\upmu}}}_{b},\mathbf{e}\right)={\mathbb{E}}\left({\alpha }_{p}{\mathrm{TM}}_{{\mathbf{e}}_{p}}\left(\mathbf{y}\mathbf{^{\prime}}\right)+{\alpha }_{c}{\mathrm{TM}}_{\mathbf{e}}\left({\mathbf{y}}\right)\right).$$

Recall that each environment $\mathbf{e}\in \mathcal{E}$ has a representation $\mathbf{e}=\left(\mathbf{d},\mathbf{v}\right)$, where vector $\mathbf{d}\in \mathcal{D}$ characterizes the owner’s desires, and vector $\mathbf{v}\in \mathcal{V}$ contains all parameters that might influence how the owner evaluates the suitability of an animal to satisfy his desires.

If the functional form of the total merit function ${\mathrm{TM}}_{\mathbf{e}}$ is known, then the breed’s adaptation to an envisaged niche ${\mathcal{E}}_{b}$ can be obtained with the Eqs. (1) and (3). The functional form of the total merit function can be modelled as follows. For an owner who provides environment $\mathbf{e}$, the total merit of animal $i$ has the representation:

$${\mathrm{TM}}_{\mathbf{e}}\left({\mathbf{y}}_{{{i}}}\right)={\phi }_{\mathbf{v}}\left({\mathbf{y}}_{{{i}}}\right)+{\lambda }_{\mathbf{e}}\left({\mathrm{R}}_{\mathbf{e}}\left({\mathbf{y}}_{{{i}}}\right)-{\mathrm{C}}_{\mathbf{e}}\left({\mathbf{y}}_{{{i}}}\right)\right),$$

where ${\phi }_{\mathbf{v}}\left({\mathbf{y}}_{{{i}}}\right)$ is the monetary profit, which the owner has from owning animal $i$, ${\mathrm{R}}_{\mathbf{e}}\left({\mathbf{y}}_{{{i}}}\right)$ is the non-monetary reward that the owner perceives he has, and ${\mathrm{C}}_{\mathbf{e}}\left({\mathbf{y}}_{{{i}}}\right)$ is the cost that the owner perceives he has in addition to the true monetary costs. An owner perceives he has high costs if he does not perceive that the breed is easy-going. The factor ${\lambda }_{\mathbf{e}}\ge 0$ weights perceived rewards and costs relative to the monetary rewards and costs. It is common to assume ${\lambda }_{\mathbf{e}}=0$ for producers, in which case the perceived rewards and costs are ignored. However, if the producers believe that their interests are not well reflected by the selection index that relies on the definition of the total merit function, then they might ignore the selection index, switch to other breeds, or stop breeding. The appropriate weight ${\lambda }_{\mathbf{e}}$ for producers is, therefore, close to zero but different from zero. The value ${\lambda }_{\mathbf{e}}=1$ could be chosen for the consumers. The non-monetary reward that an owner who provides environment $\mathbf{e}$ perceives he has from owning animal $i$ can be decomposed as:

$${\mathrm{R}}_{\mathbf{e}}\left({\mathbf{y}}_{{{i}}}\right)={w}_{\mathbf{e}1}{\mathrm{R}}_{\mathbf{e}}^{\mathrm{Perf}.}\left({\mathbf{y}}_{{{i}}}\right)+{w}_{\mathbf{e}2}{\mathrm{R}}_{\mathbf{e}}^{\mathrm{Emot}.}\left({\mathbf{y}}_{{{i}}}\right)+{w}_{\mathbf{e}3}{\mathrm{R}}^{\mathrm{Altr}.}\left({\mathbf{y}}_{{i}}\right),$$

where ${\mathrm{R}}_{\mathbf{e}}^{\mathrm{Perf}.}\left({\mathbf{y}}_{{{i}}}\right)$ is the reward that the owner perceives he has from the physical and mental performance of the animal, ${\mathrm{R}}_{\mathbf{e}}^{\mathrm{Emot}.}\left({\mathbf{y}}_{{{i}}}\right)$ is the reward that the owner perceives he has from the animal’s ability to satisfy his emotional needs and desires, and ${\mathrm{R}}^{\mathrm{Altr}.}\left({\mathbf{y}}_{{{i}}}\right)$ measures the animal’s ability to satisfy altruistic desires of the owner. The weights ${w}_{\mathbf{e}k}\ge 0$ are assumed to be statistically independent from the corresponding rewards, which is needed for the calculation of expected values.

Note that each term that contributes to the total merit of an animal corresponds to one subspace of $\mathcal{D}$ from Eq. (2). That is, each category of desires resulted in defining a different merit function. The different merit functions ${\phi }_{\mathbf{v}}$, −${\mathrm{C}}_{\mathbf{e}}$, ${\mathrm{R}}_{\mathbf{e}}^{\mathrm{Perf}.}$, ${\mathrm{R}}_{\mathbf{e}}^{\mathrm{Emot}.}$, and ${\mathrm{R}}^{\mathrm{Altr}.}$ are described below, i.e. how different desires affect owner preferences for specific animal traits, and how influencing variables might affect the way, an owner evaluates the suitability of the animal to satisfy his desires. A functional form for the merit function is proposed when appropriate, and how the parameters of the merit function could be estimated is described.

#### The merit for profit

Profitability implies that a buy-sell transaction takes place, and thus a product has to exist. In the case of a companion dog breed, the products are the puppies, while in the case of dairy cattle, the products are the milk produced, the cows culled, and the calves sold.

The profit function ${\phi }_{\mathbf{v}}\left({\mathbf{y}}_{{{i}}}\right)$ for a producer measures the profit that the producer can achieve by keeping the animal. Relevant to profit calculations is the difference between revenue and production costs per unit of the final product. When different products are sold, then the net profit of the farm per animal place and year could be considered an appropriate target quantity, which is often multiplied with the average length of productive life.

Traits from all categories can affect an animal’s profit. For example, production traits affect the revenue, functional traits affect the production costs, good mental or physical performance can increase sales proceeds from the sale of offspring, body size affects the feeding costs, and inappropriate behaviour can cause additional workload. Influencing variables from almost all the categories can affect an animal’s profit. For example, an animal’s profit is affected by the market prices of products and resources, legislative framework conditions such as taxes on methane emissions, and environmental conditions such as soil quality.

The methodology for computing an animal’s profit includes profit equations and simulations based on bio-economic modelling [17–20].

The profit function ${\phi }_{\mathbf{v}}\left({\mathbf{y}}_{{{i}}}\right)$ for a consumer who buys live animals measures the monetary profit that he has from owning the animal. If the consumer does not earn any income from his animals, then his profit is negative and equals the monetary costs that are associated with owning the animal.

#### The merit for performance

The merit of the animal for performance ${\mathrm{R}}_{\mathbf{e}}^{\mathrm{Perf}.}\left({\mathbf{y}}_{i}\right)$ aims at predicting the benefit that an owner perceives he has from the physical and mental performance of the animal. The merit function depends on the environment $\mathbf{e}=\left(\mathbf{d},\mathbf{v}\right)$ provided by the owner. As highly performing animals could overwhelm their owners, an optimum value ${\mathrm{Opt}}_{\mathbf{e}k}$ is likely to exist for each performance and behaviour trait $k$. The optimum value depends on the owner traits included in vector $\mathbf{v}$, and on the activities for which the animal should be used as described by vector $\mathbf{d}$. For example, a horse used for recreational activities is likely to have a different optimum performance than a horse used for equestrian show jumping tournaments. The merit for performance could be defined as:

$${\mathrm{R}}_{\mathbf{e}}^{\mathrm{Perf}.}\left({\mathbf{y}}_{i}\right)={\tau }_{\mathrm{max}}-\sum_{k}{\omega }_{\mathbf{e}k}\left|{\mathrm{y}}_{ik}-{\mathrm{Opt}}_{\mathbf{e}k}\right|,$$

where the sum is taken over the relevant performance and behaviour traits. Wellmann [10] shows how merit functions with this functional form can be integrated for deriving selection indices. The weights ${\omega }_{\mathbf{e}k}$ of the traits could be determined by expert assessment or by discrete choice experiments [21]. Parameter ${\tau }_{\mathrm{max}}$ is a constant that ensures that the animal’s merit is a positive number.

#### The merit for satisfying human emotional needs

Animals can provide social support by satisfying certain human emotional needs and desires. In general, the satisfaction of a human need proceeds as follows. First, a key stimulus is detected by an associated pattern recognition schema of the human brain. The detection of the key stimulus could initiate an associated behaviour of the person, and it brings the person into a positive emotional state. The key stimulus results from the fitting of the animal’s conformation or behaviour into the associated pattern recognition schema of the human brain. Hence, the satisfaction of a human need requires the animal to have a particular conformation or to show a particular behaviour.

Whether a scheme for recognising a particular pattern is inherited or acquired is not relevant for defining the merit function. What matters is the extent to which people agree upon their classifications of the animals. The recognized pattern can usually be described by an adjective or a short phrase that describes an aspect of conformation or behaviour. A pattern recognition schema might exist for any adjective that is used consistently across owners for describing a particular aspect of an animal’s appearance or personality. For example, pattern recognition schemata for appearance might exist for recognizing the strength, elegance, cuteness, balancedness, and agility of an animal.

The merit of an animal for satisfying human emotional needs and desires can be defined as:

$${\mathrm{R}}_{\mathbf{e}}^{\mathrm{Emot}.}\left({\mathbf{y}}_{i}\right)={\tau }_{\mathrm{max}}-\sum_{k}{\omega }_{\mathbf{e}k}\left|{\mathrm{y}}_{ik}-{\mathrm{Opt}}_{\mathbf{e}k}\right|,$$

where the sum is taken over all pattern recognition schemes that might be relevant. The trait value ${\mathrm{y}}_{ik}$ is the animal’s score for pattern $k$, ${\mathrm{Opt}}_{\mathbf{e}k}$ is the optimum score in the opinion of owners whose desires are described by vector $\mathbf{e}$, and ${\omega }_{\mathbf{e}k}$ is the importance that these owners place on pattern $k$.

For example, a human desire could be to use a dog as a child surrogate. The cuteness of the dog is associated with its ability to satisfy that desire of the owner. Therefore, the corresponding value ${\mathrm{Opt}}_{\mathbf{e}k}$ quantifies how cute the owner wants his dog to be, the weight ${\omega }_{\mathbf{e}k}$ measures how important this is for the owner, and ${\mathrm{y}}_{ik}$ is the cuteness score of dog $i$. The cuteness score could be obtained in practice by asking owners to score pictures of dog heads, which provides an average score for each dog head. The breed judge could then choose the picture that most closely resembles the dog he is judging and then give the dog the same score. A more sophisticated procedure could use a deep neuronal network to calculate a cuteness score of an animal from a picture or a 3D image [22–24].

This merit function could not only be relevant to companion breeds, but also to livestock breeds. A trait is likely included in the satisfaction of a human need or desire, if its economic weight, as indicated by profit calculations, does not reflect the owner’s opinion of the trait’s importance. For example, an impressive large stature and dairy type are considered desirable by Holstein breeders but have negative economic weights. They could be included in this merit function to increase the satisfaction of the owners with their animals. However, in practice, improving dairy type may not be needed because the trait could already be near its optimum.

#### The merit for altruistic desires

The merit of the animal for satisfying an owner’s altruistic desires ${\mathrm{R}}^{\mathrm{Altr}.}\left({\mathbf{y}}_{i}\right)$ aims at predicting the benefit that an owner perceives he has from adhering to the traditional breed type. There are additional altruistic desires that a person might have, but these are ignored here because they could not be linked to specific animal phenotypes. The reward that a breeder perceives he has from adhering to the traditional breed type could be measured as:

$${\mathrm{R}}^{\mathrm{Altr}.}\left({\mathbf{y}}_{i}\right)={\tau }_{\mathrm{max}}-\sum_{k}{\omega }_{{b}_{i}k}\left|{\mathbf{y}}_{ik}-{\mathrm{Opt}}_{{b}_{i}k}\right|,$$

where the sum is over traits that have changed unintentionally in the past and over traits that should be adjusted to adhere to traditional customs. Parameter ${b}_{i}$ is the breed name of animal $i$, ${\mathrm{Opt}}_{{b}_{i}k}$ is the historic optimum for breed ${b}_{i}$ for trait $k$, and ${\omega }_{{b}_{i}k}$ is the weight given to trait $k$.

For many breeds, the breeders might not feel the need to adhere to the traditional breed type, in which case function ${\mathrm{R}}_{\mathbf{e}}^{\mathrm{Altr}.}$ would be chosen constantly zero. An example for an exception is the single-coloured German Angler cattle. Historical crossbreeding with Red Holstein introduced white markings into the breed, and removing them is considered desirable by the breeders.

#### The merit for easygoingness

A breed is perceived easy-going, if complications that could arise from owning an animal could easily be avoided. Complications could arise from the workload, from inner conflicts caused by violated ethical principles, from health problems of the animal, or from a mismatch between the animal’s and the owner’s personality or fitness. The perceived easygoingness of keeping a breed depends on functional traits, on behaviour traits that could cause problems, and on conformation traits that entail additional workload. Breeders of companion breeds and livestock breeds may both prefer to put larger weights on health traits and longevity than would be optimal for a hypothetical breeder whose sole desire is profit maximization.

The extent to which problems can be tolerated by an owner depends on certain influencing variables from subspace $\mathcal{V}$, which includes owner traits. For example, a farmer with high ethical principles might avoid keeping a chicken line with a tendency to feather pecking, a dog owner could avoid keeping a breed whose coat needs to be hand-stripped, and people insisting on non-violent dog training would likely avoid keeping a breed that permanently tries to improve its tier in the social hierarchy. The perceived easygoingness is also affected by environmental conditions. For example, short stalls of dairy cattle restrict the permissible body size, and the presence of noise-sensitive neighbours could discourage people from owning dogs that bark frequently.

The perceived cost involved in keeping the animal is:

$${\mathrm{C}}_{\mathbf{e}}^{\mathrm{Easy}.}\left({\mathbf{y}}_{i}\right)=\sum_{k}\left({\omega }_{\mathbf{e}k}-{\omega }_{\mathbf{e}k}^{\mathrm{mon}}\right){\mathbf{y}}_{ik},$$

where the sum is over all traits that affect the cost of owning the animal and for which the perceived cost ${\omega }_{\mathbf{e}k}$ is larger than the true monetary cost ${\omega }_{\mathbf{e}k}^{\mathrm{mon}}$. The monetary costs ${\omega }_{\mathbf{e}k}^{\mathrm{mon}}$ of the traits can be obtained by a first order Taylor approximation of the profit function around the vector with trait means. The perceived cost ${\omega }_{\mathbf{e}k}$ of a trait equals the price that the owners would be willing to pay for a hypothetical treatment that improves the trait of the animal permanently by one unit. These prices can be determined by discrete choice experiments.

### The optimization problem

Two approaches to improving breeding goals can be conceived. The first approach aims at increasing the size of a breed’s niche by making the breed more attractive to non-owners, while the second approach aims at increasing the adaptation of a breed to its niche by increasing the satisfaction of the owners with their breed. Ideally, both approaches would be combined.

We pursue a single-breed optimization approach that focuses on increasing the validity of a breeding goal. The approach involves defining an envisaged niche ${\mathcal{E}}_{b}$ for the breed. The validity of a breeding goal depends on the number ${\mathfrak{m}}_{{t}_{1}}\left({\mathcal{E}}_{b}\right)$ of animals from the breed that live in its envisaged niche ${\mathcal{E}}_{b}$ at the end ${t}_{1}$ of the planning horizon of the breeding program and on the adaptedness ${a}_{{t}_{1}}\left({{\varvec{\upmu}}}_{b} ,{\mathcal{E}}_{b}\right)$ of these animals to their respective environments. The adaptedness function ${a}_{{t}_{1}}$ is estimated by a function ${\widehat{a}}_{{t}_{1}}$. The optimization problem involves finding the optimum intermediate breeding goal ${\dot{{\varvec{\upmu}}}}_{b}^{i}$ that maximizes the estimated adaptedness function ${\widehat{a}}_{{t}_{1}}$ on a set ${\mathcal{U}}_{b}$ of permissible breeding goals. The set ${\mathcal{U}}_{b}$ of permissible breeding goals for breed $b$ includes only breeding goals that can be achieved until time ${t}_{1}$ but excludes breeding goals that are incompatible with animal health or animal welfare. Thus, the solution ${\dot{{\varvec{\upmu}}}}_{b}^{i}$ to the optimization problem satisfies:

4 $${\widehat{a}}_{{t}_{1}}\left({\dot{{\varvec{\upmu}}}}_{b}^{i},{\mathcal{E}}_{b}\right)=\underset{{\mu }_{b}\in {\mathcal{U}}_{b}}{\mathrm{max}}{\widehat{a}}_{{t}_{1}}\left({{\varvec{\upmu}}}_{b},{\mathcal{E}}_{b}\right).$$

A breeding goal defined as the solution of Eq. (4) is valid in the sense of the Background section, only if the future size ${\mathfrak{m}}_{{t}_{1}}\left({\mathcal{E}}_{b}\right)$ of the envisaged niche is sufficiently large. The envisaged niche ${\mathcal{E}}_{b}$ should, therefore, satisfy the following condition:

(i) The expected population size after the end of the planning horizon of the breeding program remains larger than required for the long-term survival of the breed. That is:

$${c}_{b}{\mathfrak{m}}_{t}\left({\mathcal{E}}_{b}\right)\ge {N}_{\mathrm{min}},$$

where ${c}_{b}$ is the expected proportion of animals from the envisaged niche that belong to breed $b$ at time $t\ge {t}_{1}$, and ${N}_{\mathrm{min}}$ is the minimum population size that would be required for long-term survival of a breed.

Note that a sufficient population size can only be achieved if the envisaged niche is sufficiently narrow so that the breed can perform well in all environments. Thereby, the width of a niche refers to the diversity of environments that can be tolerated by the breed [25].

The set of permissible breeding goals ${\mathcal{U}}_{b}$ belongs to the set $\mathcal{P}$ of potential breeding goals. What this set actually is, depends on the underlying mathematical model. In the simplest case, we may assume that the genetic and phenotypic covariance matrices do not change over time and that the phenotypic distribution does not depend on the environment. In this case, ${{\varvec{\upmu}}}_{b}\in \mathcal{P}$ is the vector with trait means, so $\mathcal{P}\subset {\mathbb{R}}^{K}$. More generally, the parameter ${{\varvec{\upmu}}}_{b}$ might depend on the environment $\mathbf{e}$ in which the animals are kept, in which case ${{\varvec{\upmu}}}_{b}:\mathcal{E}\to {\mathbb{R}}^{K}$ is a function. Then, the space $\mathcal{P}$ of potential breeding goals is the set of all these functions.

**Fig. 3:**
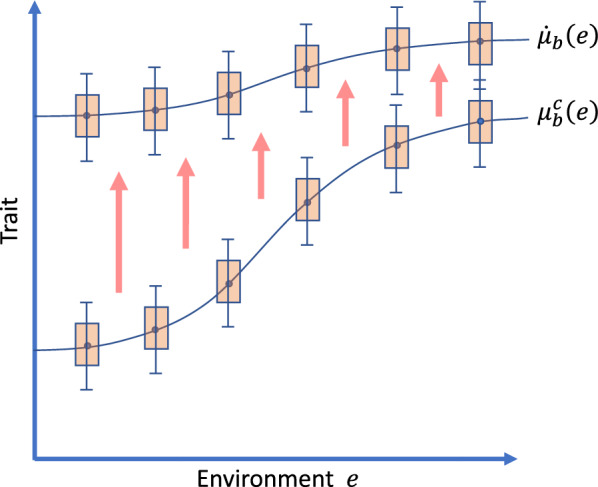
Illustration of the state space of breeding programs. Illustrative example with a one-dimensional space $\mathcal{E}$ of environments, and a one-dimensional space $\mathcal{Y}$ of phenotypes, so there is only one trait under selection. The current state of the breeding program for breed $b$ is described by a function ${\mu }_{b}^{c}\in \mathcal{P}$ that provides for each environment $e$ the trait mean of the breed in that environment. The phenotypic variance is assumed to be constant. The breeding goal of the breed is defined by function ${\dot{\mu }}_{b}\in \mathcal{P}$. The red arrows symbolize that the trait mean ${\mu }_{b}^{c}\left(e\right)$ is expected to approach the breeding goal ${\dot{\mu }}_{b}\left(e\right)$ in the course of the breeding program for all relevant environments $e$

Figure 3 illustrates how genotype-by-environment interactions could cause the phenotypic distribution to depend on the environment. For example, dairy cattle tend to have a lower milk yield on farms that are located in regions with a low soil quality, and dogs tend to show more inappropriate behaviour if their owners are inexperienced. It could be appropriate, at first reading, to consider only the important special case that ${{\varvec{\upmu}}}_{b}$ is equal to the vector with trait means and that this vector does not depend on the environment. In this case, the functions plotted in Fig. 3 would be horizontal lines.

The long-term breeding goal of breed $b$ is defined by the state ${\dot{{\varvec{\upmu}}}}_{b}\in \mathcal{P}$ that maximizes the breed’s adaptatedness to the envisaged niche on the set of potential breeding goals that could be reached before the genetic variance in the selection index disappears. The breeding goal would ideally not only specify the desired average performance, conformation and behaviour of a breed, but also how the traits should be expressed in different environments. However, in practice, only an intermediate breeding goal ${\dot{{\varvec{\upmu}}}}_{b}^{i}$ needs to be identified. The final state of the breeding program does not need to be known in advance.

### Performing the optimization

**Fig. 4:**
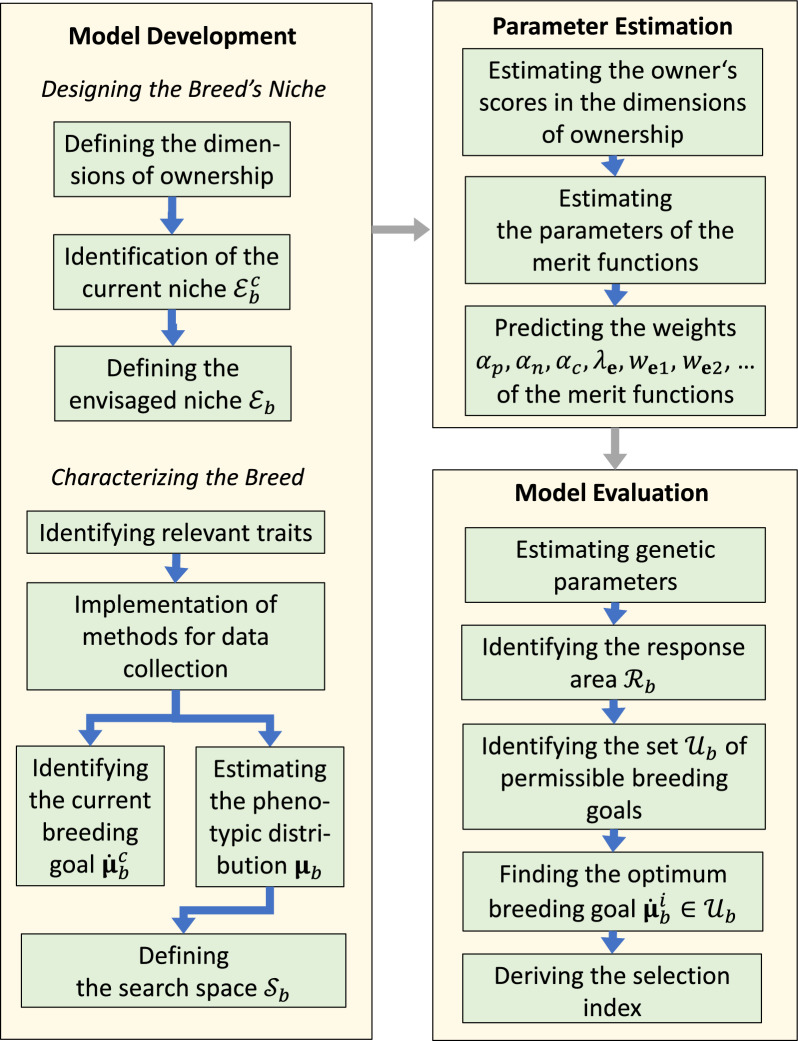
Illustration of the proposed method for breeding goal optimisation

Optimization of the breeding goal involves defining a planning horizon for the breeding program and defining an envisaged niche ${\mathcal{E}}_{b}$ for the breed that is expected to satisfy the above constraint (i). The next step is to define a search area ${\mathcal{S}}_{b}$ that includes the set ${\mathcal{U}}_{b}$ of permissible breeding goals and to estimate the adaptedness function ${a}_{{t}_{1}}\left({{\varvec{\upmu}}}_{b} ,{\mathcal{E}}_{b}\right)$ on the search area. Then, an intermediate breeding goal ${\dot{{\varvec{\upmu}}}}_{b}^{i}$ is obtained as the solution of Eq. (4). In the special case that the parameter of the phenotypic distribution is defined as the vector with trait means, the vector with the desired gains in the traits equals $\Delta {\varvec{\upmu}}={\dot{{\varvec{\upmu}}}}_{b}^{i}-{{\varvec{\upmu}}}_{b}^{c}$, where ${{\varvec{\upmu}}}_{b}^{c}$ is the vector with current trait means. The selection index can then be computed with the desired gain approach [8]. The more general case that the phenotypic covariance matrix changes over time is considered in [10]. The proposed optimization process outlined above consists of three steps, which are model development, parameter estimation, and model evaluation. These steps, which are illustrated in Fig. 4, are detailed below.

#### Model development

The mathematical model includes a definition of the breed’s envisaged niche ${\mathcal{E}}_{b}\subset \mathcal{E}$, and the definition of the search area ${\mathcal{S}}_{b}\subset \mathcal{P}$, which is the set of all putative breeding goals that are to be taken into account. The search area should be defined large enough such that it includes the set ${\mathcal{U}}_{b}$ of permissible breeding goals. In addition, the model includes equations for calculating an estimate ${\widehat{a}}_{{t}_{1}}\left( \cdot ,{\mathcal{E}}_{b}\right)$ of a breed’s adaptedness to the envisaged niche ${\mathcal{E}}_{b}$ on the search area ${\mathcal{S}}_{b}$.

Developing a mathematical model that is tailored to the breed of interest requires a thorough understanding of the breed’s current niche and its current phenotypic distribution. The first step is, therefore, the identification of the breed’s current niche ${\mathcal{E}}_{b}^{c}$. Second, the breed’s envisaged niche ${\mathcal{E}}_{b}$ is defined. It can be chosen equal to the current niche, except if the breed’s current niche violates condition (i). Third, the traits of the breed are characterized. Fourth, the data that were collected on the traits are used for defining the search area ${\mathcal{S}}_{b}$. Finally, the model equations are derived. As the model equations have already been described in previous sections, they are not discussed in this section. The other steps are described below.

*Step 1: Niche characterization* For most breeds, the envisaged niche can be chosen identical to the breed’s current niche, so the first step of the optimization is the identification of the breed’s current niche. The breed’s current niche is defined by a description of the desires that are typical of what the owners associate with owning the breed, and by the ranges of influencing variables.

For example, the main desires that farmers associate with owning animals are usually that the animals should contribute to their income and that they are easy-going. In contrast, the main desires of companion animal owners are often that the animals should satisfy specific emotional needs, i.e. that they are easy-going, beautiful, and that they should be suitable for certain leisure activities.

The desires of typical owners can be identified by determining the owner’s scores in the different dimensions of ownership by questionnaires. For determining the values of influencing variables, market prices can be obtained by market analyses, while owner traits and the physical environment can be identified by questionnaires.

*Step 2: Niche design* The envisaged niche ${\mathcal{E}}_{b}$ of a breed characterizes the desires of people who are expected to be typical future owners of the breed and provides the ranges of influencing variables. For defining the envisaged niche, we start with a superset ${\mathcal{E}}_{b}^{*}$ of the breed’s current niche ${\mathcal{E}}_{b}^{c}$. The set includes characterizations of owners of the breed, but also characterizations of people who currently do not own the breed but might decide to do so after the breed has been improved. This set can often be partitioned into disjoint subniches ${\mathcal{E}}_{ub}^{*}$, so:

$${\mathcal{E}}_{b}^{*}=\bigcup_{u\in {U}^{*}}{\mathcal{E}}_{ub}^{*}.$$

The different subniches correspond to people who keep their animals for different reasons, or to physical environments with strong genotype-by-environment interactions. In both cases, it might be difficult for a breed to satisfy the desires of all owners simultaneously. The breed’s envisaged niche ${\mathcal{E}}_{b}$ should consist, therefore, of appropriately chosen subniches ${\mathcal{E}}_{ub}^{*}\subset {\mathcal{E}}_{b}^{*}$. A subniche ${\mathcal{E}}_{ub}^{*}$ should be excluded from the envisaged niche if ${\mathfrak{m}}_{t}\left({\mathcal{E}}_{ub}^{*}\right)\stackrel{t\to \infty }{\to }0$, which means that the number of owners who provide that subniche will decline severely in the future. Such subniches can be called endangered. Historically, endangered subniches were provided by dog owners who kept their dogs for dog fights or as turnspit dogs, and by farmers who kept their cattle and horses as draft animals.

Often, more than one domestic breed has been adapted to a niche. For example, various cattle breeds exist that are all kept for milk production. This is not necessarily a problem as several domestic breeds can coexist in the same niche. Nevertheless, in such cases it can make sense to adapt the different breeds to different subniches in order to facilitate adaptive radiation. That is, the niche partitioning should be coordinated with breeding organizations of other breeds in order to achieve satisfactory population sizes for all breeds.

*Step 3: Trait characterization* Traits are identified that are potentially relevant for future owners. For livestock breeds, all traits are relevant that affect the breed’s profit. However, the relevance of many other traits is not obvious but can be derived from the characteristics of the breed’s envisaged niche and from owner preferences. For example, as companion dogs are often kept as child surrogates for satisfying care-giving behaviour, many owners prefer dogs who fit the small child pattern, i.e., are cute [26]. In this case, the cuteness of the animal would be considered a trait.

A list of potentially relevant traits is created, methods for collecting data on these traits are implemented, and trait means and phenotypic variances are estimated. Whenever possible, trait heritabilities and genetic correlations should also be estimated at this stage.

In addition, the breed’s current breeding goal ${\dot{{\varvec{\upmu}}}}_{b}^{c}\in \mathcal{P}$ should be identified. As breeders and breeding organizations have often already dealt with what the optimum breeding goal might be, knowledge of the current breeding goal helps identifying the relevant traits. The current breeding goal is also needed for evaluating the success of the optimization in retrospect.

*Step 4: Defining the search area* Once the relevant traits are identified, and their genetic parameters have been estimated, the search area can be defined. The search area ${\mathcal{S}}_{b}\subset \mathcal{P}$ is the area to which the adaptedness function ${a}_{{t}_{1}}\left( \cdot ,{\mathcal{E}}_{b}\right)$ will be fitted. It should include the response area ${\mathcal{R}}_{b}\subset {\mathcal{S}}_{b}$ of the breed, but exclude putative breeding goals that are incompatible with animal health or animal welfare.

The response area ${\mathcal{R}}_{b}$ consists of all putative breeding goals that could be reached until the end of the planning horizon of the breeding program [6, 7]. Thus, the response area is determined by the planning horizon. Although the planning horizon should be sufficiently long such that genetic progress can be made, a long planning horizon could reduce the index weights of important traits. It seems, therefore, appropriate to choose the planning horizon no longer than (say) 20 years.

**Fig. 5:**
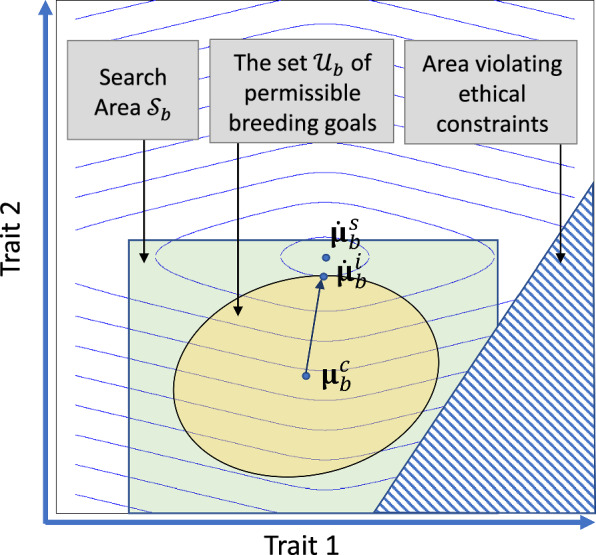
Illustration of the set of permissible breeding goals. Illustrative example with a two-dimensional space of phenotypes. The level sets of the objective function indicate the position of the optimum breeding goal ${\dot{\varvec{\upmu} }}_{b}^{s}$ in the search area ${\mathcal{S}}_{b}$ for the breed. The search area includes the response area of the breed but excludes putative breeding goals that violate ethical constraints. The set ${\mathcal{U}}_{b}$ of permissible breeding goals is included in the response area. The optimum permissible breeding goal ${\dot{\varvec{\upmu}}}_{b}^{i}$ has the property that the level sets of the objective function are tangential to the boundary of ${\mathcal{U}}_{b}$. The vector from the current trait means ${\varvec{\upmu}}_{b}^{c}$ to the optimum permissible breeding goal ${\dot{\varvec{\upmu}}}_{b}^{i}$ defines the optimum selection index

When the phenotypic covariance matrix is assumed not to change over time, then, for each environment, the response area is an ellipsoid with centre equal to the vector of current trait means. The shape of the ellipsoid can be determined when the heritabilities and genetic correlations are known. Consequently, when these parameters have already been estimated, then the search area can be restricted to the response area. Unfortunately, however, these parameters are often unknown at the time when the optimization should be carried out. In that case, it would be convenient to define the search area as the set of putative breeding goals for which all trait means deviate less than (say) two phenotypic standard deviations from the vector with current trait means. A pragmatic approach would then be to define the planning horizon such that the response area ${\mathcal{R}}_{b}$ fits into the search area ${\mathcal{S}}_{b}$. A possibility to define the search area is illustrated in Fig. 5.

Putative breeding goals are excluded from the search area if they are incompatible with animal health or animal welfare. This could comprise, for example, chicken with a tendency to feather pecking, wire-haired dogs with coats that need to be hand-stripped, or dogs with very deep-set eyes as they tend to have eye-lid problems. Care must be taken that several traits are not incompatible with animal welfare, but are genetically correlated with such traits. Examples include the milk yield of dairy cattle and the muzzle length of dogs. It is not appropriate to ban high milk yield or short muzzles from the breeding goal. Instead, it needs to be ensured that the breeding goal asks for the absence of the correlated health problems. This includes, for example, the presence of ketosis in dairy cattle and the brachycephalic obstructive airway syndrome in dogs.

The set ${\mathcal{U}}_{b}$ of permissible breeding goals is then obtained by restricting the search area to the response area, i.e., ${\mathcal{U}}_{b}={\mathcal{S}}_{b}\cap {\mathcal{R}}_{b}$.

#### Parameter estimation

Procedures for estimating the merit functions need to be implemented. The profit function ${\phi }_{\mathbf{v}}$ can be computed with profit equations, the perceived costs ${\mathrm{C}}_{\mathbf{e}}$ can be obtained by estimating the owner’s willingness to pay for trait improvements, and the merit functions ${\mathrm{R}}_{\mathbf{e}}^{\mathrm{Perf}.}$, ${\mathrm{R}}_{\mathbf{e}}^{\mathrm{Emot}.}$, and ${\mathrm{R}}^{\mathrm{Altr}.}$ can be also estimated with stated preference techniques such as contingent valuation and discrete choice experiments [27] or hill-climbing algorithms [28]. Because discrete choice experiments can handle only a limited number of attributes at a time, the parameters of the different merit functions should be estimated in different discrete choice experiments. In a last step, the expected values of the weights ${\alpha }_{p},{\alpha }_{n},{\alpha }_{c} ,{\lambda }_{\mathbf{e}},{w}_{\mathbf{e}1},{w}_{\mathbf{e}2},\dots$ of the different merit functions are estimated in a discrete choice experiment. A stated preference technique seems appropriate for the final step because the importance of different breeding objectives can usually not be compared in monetary terms. The participants of the study should be breeders and owners who provide the envisaged niche for their animals. The traits of the animals that are to be evaluated should be approximately evenly distributed in the search area ${\mathcal{S}}_{b}$. The search area could be narrowed down in a second evaluation in order to get a more accurate estimate of the total merit function in the vicinity of its optimum. Once all surveys are finished, an estimate ${\widehat{a}}_{{t}_{1}}\left({{\varvec{\upmu}}}_{b},{\mathcal{E}}_{b}\right)$ of the objective function for any putative breeding goal ${{\varvec{\upmu}}}_{b}\in {\mathcal{S}}_{b}$ is available.

#### Model evaluation

The optimum breeding goal in the search area is not necessarily a permissible breeding goal as it could be outside the response area. Therefore, the local maximum ${\dot{{\varvec{\upmu}}}}_{b}^{i}$ of the estimated objective function ${\widehat{a}}_{{t}_{1}}\left( \cdot ,{\mathcal{E}}_{b}\right)$ in the set ${\mathcal{U}}_{b}\subset {\mathcal{S}}_{b}$ of permissible breeding goals needs to be identified, which could be done by a hill-climbing algorithm or another optimization algorithm.

The set of permissible breeding goals is restricted by the response area ${\mathcal{R}}_{b}$, which in turn is determined by the genetic parameters and the selection intensity. Thus, the model evaluation starts with estimating the trait heritabilities and genetic correlations. The estimates are used for identifying the response area ${\mathcal{R}}_{b}$ and the set ${\mathcal{U}}_{b}$ of permissible breeding goals.

Then, the local maximum ${\dot{{\varvec{\upmu}}}}_{b}^{i}\in {\mathcal{U}}_{b}$ of the objective function is determined which is the new intermediate breeding goal. According to [6], the optimum selection index is then defined by the straight line from the vector ${{\varvec{\upmu}}}_{b}^{c}$ with current trait means to the local maximum ${\dot{{\varvec{\upmu}}}}_{b}^{i}$, and can be computed with the desired gain approach [8].

The closeness of the result to the solution of the optimization problem depends on how accurately future owners of the breed can be characterized, on the accuracy with which function ${a}_{{t}_{1}}\left( \cdot ,{\mathcal{E}}_{b}\right)$ could be estimated, and on the standard errors of the phenotypic and genetic covariances.

## Example

Different approaches to breeding goal optimization are compared with the example of an arbitrary dog breed that is primarily kept for companionship. These approaches are:

1. Expert assessments.
2. Profit maximization.
3. The proposed approach.

### Expert assessments

The current breeding goals of dog breeds are defined by their breed standards, which are based on expert assessments. Many characteristics are included because the dogs that founded the breed possessed them. Breed standards usually intend to describe trait characteristics that allow a dog to do the work he was originally intended for, rather than the work that the breed is expected to do in the future. Breed experts often argue that breeders should not breed for today’s fashion. Instead of breeding dogs that fit into today’s society, they should keep the instincts of the dogs alive and give them the chance to fulfil the purpose for which they evolved [29]. The problem here is that many breed experts have the altruistic desire to cling to tradition and dismiss the desires of consumers as secondary. Hence, expert assessments can provide valid breeding goals, but this is not the most likely outcome because many experts do not know what constitutes a valid breeding goal. Although many kennel clubs are now conducting behaviour tests and genetic tests, the introduction of behaviour tests was often prompted by societal pressures rather than the breed experts' own initiative.

### Profit maximization

Profit maximization provides the economic weights of the traits. The economic weight of a trait specifies the change in economic outcome of a breeder that is caused by a change in the genetic value of the trait. The products that are produced and sold are the dog puppies. A breeder can increase his income by breeding dogs that can be sold at higher prices, or by reducing the production costs. The production costs per puppy can be reduced by breeding for good fertility, sufficient milk yield, good maternal instincts, and low stillbirth rates, but also by breeding for small body size to reduce feeding costs. Although these could be reasonable points, improving these traits does not make the breed more suitable for its task of providing social support for its owners.

Puppies are often sold at different prices. Puppies with faults that result in veterinary costs (e.g. retained testicles) are often sold at lower prices, while show-quality puppies that are primarily sold to other breeders can be more expensive. The breeder can thus increase his income by specializing on breeding show-quality puppies. These puppies, which are often sold at a later age, are close to the breed standard. Hence, the result of profit maximization is that the trait characteristics described in the breed standard are made more extreme.

It might be possible for a breeder to take higher prices for dogs with characteristics that are much thought-after by non-breeders and to breed explicitly for those traits, but this is seldom done. Breeders might not do this because they cannot prove that their puppies possess the claimed characteristics, or because their dogs would not be eligible for breeding if they do not look or behave ‘typical’ for their breed. Consequently, profit maximization does not result in a valid breeding goal, but results in the confirmation of wrong beliefs.

### The proposed approach

The proposed approach first defines a target group of owners, which are the owners who provide the envisaged niche for the breed. Then, an inventory is made of the desires that these people associate with owning a dog. It is then predicted whether this group of owners remains sufficiently large to ensure the long-term survival of the breed. It could be predicted from the desires stated by the owners regarding which trait characteristics they are likely to prefer. Then, the true preferences of these people are determined by carrying out discrete choice experiments. The discrete choice experiments are used for identifying trait optima and trait importance, which provides an estimate of the adaptedness function. The trait optima are then used to revise the breed standard, and the estimated adaptedness function is used for identifying the desired gains of the traits. Finally, the selection index is determined with the desired gain approach. This approach is likely to result in a valid breeding goal because it explicitly takes consumer preferences into account, which could subsequently cause the population size to increase.

This example demonstrates that the established methods for breeding goal optimization all fail to provide valid breeding goals for companion breeds, while the proposed approach has the potential of making substantial progress in dog breeding. Without the proposed approach, people would generally not be able to know how the vector with desired gains needs to be defined, and whether certain trait characteristics are desirable or undesirable. In addition, companion dogs have a huge number of traits that might be relevant. The proposed approach identifies the important ones and neglects the others.

## Discussion

A general framework for the definition of valid breeding goals of animal breeds is proposed. A breeding goal was called valid, if there exists a sufficiently large niche for a breed with the envisaged phenotypic distribution. Therefore, the proposed approach to breeding goal optimization consists of defining an envisaged niche for the breed, and of finding the optimum performance, behaviour, and conformation of the breed that maximizes the adaptedness of the breed to its envisaged niche. The definition of the envisaged niche consists of a characterization of owners that are expected to keep the breed in the future, while the adaptedness of the breed to its niche predicts the satisfaction of these future owners with the breed.

### Relevance of the approach

Various methods have been proposed in the literature for deriving index weights for non-economic traits, which include: accounting only for the economic consequences of undesired trait expressions [30], determining the farmer's willingness to pay for trait improvements [21], applying the desired gain approach to an intuitively defined intermediate breeding goal, and deriving restricted selection indices that ensure that the non-economic traits do not get worse [31]. All these approaches have in common that they do not explicitly take the multiple desires that people associate with owning an animal into account.

The traditional approach to breeding goal optimization according to [5] considers only the monetary desires of the owners. The paper pointed out that owners have additional desires that should be satisfied, which includes, among others, the desire for easygoingness, and the desire to be surrounded by good-looking animals. The total merit of an animal should, therefore, be composed of different merit functions. One of them could measure the monetary profit of the animal, one its easygoingness, and one its aesthetic value. Easygoingness is improved by the absence of health problems, so the implementation of this approach in practice would also be beneficial to animal welfare.

For companion breeds, the owner’s monetary desires are negligible, and the desire for easygoingness can never be the only reason for owning an animal. This is because it would be most easy-going to own no animal at all. Consequently, breeding goal optimization has to focus on the breed’s ability to satisfy human emotional needs. The optimization of breeding goals of companion breeds has long been hampered by the lack of a scientific framework by which to perform the optimization. The present paper provides an appropriate framework. Although the breeding goals of many dog breeds have recently been altered to account for possible health problems, the validity of their breeding goals is still in question. Their validity is in question because the breeding goals of many dog breeds are defined by having their historic rather than their future uses in mind, or simply by describing how an ordinary specimen of the breed looked. This practice does not threaten the validity of a breeding goal if the owners primarily keep the breed for satisfying their altruistic desire of adhering to traditional customs, or if the favoured trait characteristics correlate with owner preferences by chance. Checking and revising the breeding goals of certain animal breeds with the aim to improve their validity is work that still needs to be done.

### Consumer interests

The consumers are the people who buy the final products, which are the pork in the case of pig breeding and the dog puppies in the case of companion dog breeding. When consumers pay for improved product quality, then there is no doubt that including product quality in the breeding goal increases its validity. However, it is less obvious, whether breeding for improved product quality is appropriate when consumers would appreciate it, but do not pay for it. Examples are increasing the meat quality of pork, or reducing behaviour problems of companion dogs. Improved product quality is likely to increase the demand for the product, which in turn increases product prices. Higher product prices would cause the breeders to keep more animals, which increases the population size. As selection towards improved product quality increases the future population size of the breed, this contributes to the validity of the breeding goal. Consequently, breeding for improved product quality always increases the validity of a breeding goal, regardless of whether consumers pay for it or not.

### Public interests

The proportion of household spending on food in Germany has fallen from 61% in 1850 to 15% in 2021 (Statistisches Bundesamt), which implies that the interest of the public in further optimizing the productivity of the food chain decreases. The decreasing marginal return of additional food production to the farmer caused animal breeders to focus on the cost side, which can help to increase the health and longevity of livestock breeds [32]. The farmers are increasingly faced with newly-emerged desires of the public, such as reducing greenhouse gas emissions, improving animal welfare, promoting biodiversity, renaturation, and small-scale farming. Accounting for such desires requires scientists to obtain a thorough understanding of what makes up a valid breeding goal, and to engage in advising politicians how the legislation can facilitate achieving these goals by providing incentives and deterrents that canalize the interests of the farmers.

For example, the public has an interest in reducing greenhouse gas emissions. Cattle breeders might be able, in the future, to contribute to this goal by including a breeding value for greenhouse gas emissions in the selection index [33]. This might increase or decrease the validity of the breeding goal, depending on the legislation. To see this, remember that the validity of a breeding goal is determined by the ability of the breed to satisfy the desires of the owners, which in turn depends on the values of influencing variables. Cattle breeders themselves rarely have an own interest in keeping cows with reduced greenhouse gas production. Moreover, the greenhouse gas production of a cow is currently not directly associated with any variable from the space $\mathcal{V}$ of influencing variables that affects the breed’s profit. If this were to remain so in the future, then breeding for reduced greenhouse gas emissions would compromise the validity of the breeding goal because it reduces the genetic progress that can be achieved for other traits. Consequently, altering a breeding goal with the aim to account for the interests of second parties usually requires the introduction of legislative framework conditions, the purpose of which is to canalize the interests of the owners. Such laws would harness the self-interest of the owner for the benefit of second parties. A careful design of these laws is especially important because of the large delay between the start of a breeding program and the achievement of the breeding goal. On the one hand, the incentives or deterrents need to be sufficiently severe for affecting an owner’s behaviour, but on the other hand, they must not cause a breakdown of the population size. In the case of greenhouse gas emissions, emission allowance trading might be the method of choice. As the greenhouse gas emission of a cow depends on its hypothetical breeding value for greenhouse gas emissions, it would depend on the costs of emissions certificates, on the phenotyping costs, and on the genetic correlations with other traits, whether including greenhouse gas emissions in the breeding goal increases or decreases its validity.

### Animal welfare

Not only the public, but also the animals themselves have interests that might be in conflict with the interests of the owners. In particular, animal health and animal welfare should be maintained in a breeding program. The proposed approach to optimization of the breeding goal accounts for animal health and animal welfare in two ways. First, trait expressions that would be incompatible with animal health or animal welfare are excluded from the set of permissible breeding goals, and second, positive index weights would be given to health traits. Health traits would be included in the selection index because poor animal health reduces the easy-goingness of a breed, and it can also reduce the monetary profit of the breed.

The approach of accounting for animal health and animal welfare can be explained at the example of a breeding company that has a breeding line with a trait that makes their product come below an acceptance threshold (e.g., high mortality). Such a breeding line is at risk of losing market access. In the case that the problem is related to animal health or animal welfare, this implies that the current parameter ${{\varvec{\upmu}}}_{b}^{c}$ of the phenotypic distribution is outside the set ${\mathcal{U}}_{b}$ of permissible breeding goals. Such a situation arises most likely because the breeding line had been selected for important correlated traits. The planning horizon for solving this problem should be chosen short, but sufficiently long such that the set of permissible breeding goals is non-empty and contains points that provide acceptable values for the correlated traits. Then, the new intermediate breeding goal ${\dot{{\varvec{\upmu}}}}_{b}^{i}\in {\mathcal{U}}_{b}$ is identified that maximizes the adaptedness function, and the selection index is obtained with the desired gain approach. The selection index would then prompt the breeding company to focus mainly on the single trait until the problem is solved and they can ensure market access. On the other hand, if a health trait is insufficient but market access is not at risk, then a positive index weight would be given to the health trait. The index weight depends on the importance farmers place on the health and easy-goingness of their animals, and on the effect the trait has on the profit of the breed. In any case, the index weights for health traits should be chosen sufficiently large so that the health improves over time.

Because owners are usually unhappy if their animals are not healthy, there is no fundamental conflict between the interest of the animal to be healthy and the interests of the owner. Nevertheless, various veterinarians suspect that certain animal breeds, such as the Pug dog breed [34], and the Holstein cattle [35] are so-called torture breeding animals, which, according to the German animal protection law, (“Qualzuchtparagraph”), would imply that keeping these breeds has to be banned in Germany. This paper emphasized that animal breeds are never bred to suffer. They are always bred to satisfy certain human needs and desires, which was the reasonable ground for the breeds to come into existence. Although no one has an interest in breeding unhealthy animals, some domestic breeds indeed have health problems. It needs to be clarified where the health problems come from and how they can be handled. Veterinarians often suspect that the reasons for health problems are inappropriate breeding goals [34, 35], but this is rarely the case. There are notable exceptions, such as the Pekingese dog breed, whose excessive coat can lead to heat stress in the summer. Other examples could be dog breeds with a long rough coat that can only be maintained if the groomers regularly pull-out the hairs by hand. The breed standards of many dog breeds have been revised to avoid welfare issues [36, 37]. However, more often the reason for the exaggeration of a trait were ambiguous breed standards. An example is the breed standard of the pug that, until recently, asked for a short muzzle without saying how short “short” is. Therefore, it can be recommended to augment the breed standards with sculptures that unambiguously define the desired trait expressions. Metric traits that are suitable for breeding value estimation can then be extracted from the sculptures. Another frequent reason for a health problem is that a desired trait expression is genetically correlated with health traits, but the breeding organisations and veterinarians failed to establish a comprehensive data base with health records that is suitable for breeding value estimation. For example, many people favour dogs with short muzzles because they fit into the small child pattern. However, the health problems of the breeds did not arise because people favour dogs with short muzzles, but because an insufficient selection intensity has historically been placed on the associated health traits. A further reason for health problems can be inbreeding depression, in which case the genetic variation of the population should be increased.

Although the situation in dog breeding has improved in recent years because health screenings are now being implemented, and the situation in dairy cattle breeding has improved in Germany because health traits and the length of productive life are now included in the selection index, some time will pass until a breed shows a sufficient response to an altered breeding program. Impermissible claims that a breeding program fails often come from the false belief that a breeding program can be evaluated by looking at a breed at a single point in time. However, it is not scientifically permissible to draw conclusions about a breed from the phenotypic relationships between high- and low-performing animals, or long- and short-muzzled dogs, in the event of a genetically caused improvement in the associated health traits [38].

### Crossbreeding

Although the framework for breeding goal optimization was developed for pure breeding, it can be applied to cross-breeding as well, but then the terms in Eq. (3) have a different interpretation. For example, consider a pig breeding company that breeds the parental lines of a terminal cross. The breeding company wants to derive index weights for a parental line. In this case, breed $b$ would be the parental line of interest, and environment ${\mathbf{e}}_{p}$ would be the environment provided by the breeding company. As the breeding company is expected to breed for profit, the total merit ${\mathrm{TM}}_{{\mathbf{e}}_{p}}\left(\mathbf{y}\mathbf{^{\prime}}\right)$ of an animal from the parental line for the breeding company would be equal to its monetary total merit. Moreover, ${\mathrm{TM}}_{\mathbf{e}}\left({\mathbf{y}}\right)$ is the total merit of a crossbred animal for a farmer, and $\mathrm{PQ}\left(\mathbf{y}\right)$ is the quality of the pork produced by the farmer. Then, the adaptedness function depends on the trait means ${{\varvec{\upmu}}}_{b}\left(\mathbf{e}\right)$ of crossbred animals, and on the trait means ${{\varvec{\upmu}}}_{b}\left({\mathbf{e}}_{p}\right)$ of animals from the parental line.

### Outlook

A framework was developed to provide a sound scientific basis for optimizing the breeding goals of breeds that are not solely kept for profit. Although the framework could be especially suitable for optimizing the breeding goals of companion breeds, it can also be applied to livestock breeds, such as cattle and pigs. In particular, it is suggested to compute merits for easygoingness and beauty in addition to the merit for profit, and to account for them in the selection index. For livestock, improving easygoingness would translate to placing index weights on functional traits and behaviour traits, such as, mothering ability or aggressiveness in pigs that exceed their economic values. The phenotypic distribution of a breed depends, in general, on the set of environments in which it is kept. Further work is needed to work out how this observation can be best integrated into procedures for deriving selection indices.

## Conclusions

A general approach for characterizing and defining valid breeding goals is proposed. The approach consists of defining an envisaged niche for a breed and of identifying the optimum performance, conformation and behaviour that optimizes the adaptation of the breed to its envisaged niche. Owners provide niches for their breeds in the sense that they define the conditions to which breeds may adapt and are simultaneously the resources for which the breeds compete. The proposed approach enables the optimization of breeding goals of breeds for which profit maximization is not the sole objective, and it allows to account for both, the interests of producers and consumers. It was shown using the example of companion breeds that conventional approaches to breeding goal optimization fail when consumer preferences are not well reflected by the product prices or when the breeders do not breed for profit. Optimizing the ability of different breeds to match the desires of different owners can lead to a phenotypic diversification of the breeds and to an improved adaptation of a domestic species to the environments in which it can exist.

## Data Availability

Not applicable.
